# Factors associated with serum fetuin-A concentrations after long-term use of different phosphate binders in hemodialysis patients

**DOI:** 10.1186/s12882-016-0245-3

**Published:** 2016-03-23

**Authors:** Hsin-Hung Lin, Hung-Hsiang Liou, Ming-Shiou Wu, Chiu-Ching Huang

**Affiliations:** The Kidney Institute and Division of Nephrology, China Medical University Hospital, Taichung, Taiwan; Graduate Institute of Clinical Medical Science, College of Medicine, China Medical University, Taichung, Taiwan; Hsin-Jen Hospital, Taipei, Taiwan; Division of Nephrology, National Taiwan University Hospital, Taipei, Taiwan

**Keywords:** Phosphate binder, Sevelamer, Fetuin-A, Hemodialysis, CKD, ESRD

## Abstract

**Background:**

Fetuin-A is known as a circulating inhibitor of vascular calcification. Factors associated with serum fetuin-A concentrations after long-term use of different phosphate binders in hemodialysis patients is still uncertain.

**Methods:**

In the post-hoc study, we analyzed serum fetuin-A and biochemical factors (Ca, P, i-PTH, hsCRP, TG, LDL-C) in 50 hemodialysis patients, who completed a 48-week, open-Label, controlled randomized parallel-group study. 23 patients received sevelamer and 27 patients received calcium carbonate.

**Results:**

After the 48-week treatment, the sevelamer group had less serum calcium increment, less iPTH decrement, more ALK-P increment, more hsCRP decrement and more LDL-C decrement. There was no significant difference in the serum fetuin-A decrement between two groups. Decreased serum fetuin-A levels were found after 48-week treatment in both groups: from 210.61 (104.73) to 153.85 (38.64) ug/dl, *P* = 0.003 in sevelamer group, from 203.95 (107.87) to 170.90 (58.02) ug/mL, *P* =0.002 in calcium group. The decrement in serum fetuin-A (Δfetuin-A) levels was associated with ΔCa (*ρ* = - 0.230, *P* = 0.040), ΔiPTH (ρ = 0.306, *P* = 0.031) and Δalbumin (*ρ* = 0.408, *P* = 0.003), not associated with sevelamer use, ΔP and ΔhsCRP.

**Conclusion:**

After long-term sevelamer or calcium carbonate treatment, both groups of maintenance HD patients had lower serum fetuin-A levels. Serum levels of increased calcium, decreased iPTH and decreased albumin were associated with the serum fetuin-A decrement.

## Background

Chronic kidney diseases - mineral and bone disorders (CKD - MBD) including hyperparathyroidism, hypercalcaemia and hyperphosphataemia contribute to the development of vascular calcification and cardiovascular disease (CVD) [1–3]. Fetuin-A, a circulating inhibitor of vascular calcification, is associated with lower cardiovascular calcification and mortality in hemodialysis (HD) patients [4–7]. Fetuin-A is a reverse acute phase reactant, similar to serum albumin and other hepatic proteins and is also down regulated during inflammation [8]. It is abundant in the plasma and mainly produced by the liver in adults but its serum level is lower in advanced CKD and dialysis patients [9]. Fetuin-A mediates the formation of stable colloidal mineral–protein complexes called calciprotein particles (CPPs) [10]. Thus, fetuin-A is important in the stabilization and clearance of amorphous mineral precursor and acts as efficient barrier to slow down mineralization [11].

Oral phosphate binder plays an important role in the management of hyperphosphatemia of CKD-MBD. The non-calcium phosphate binder, sevelamer hydrochloride, has been shown to reduce progression of vascular calcification and mortality in CKD or end-stage renal disease (ESRD) patients in comparison with calcium-based phosphate binders [12–15]. Besides reducing hyperphosphatemia without calcium loads, sevelamer has pleiotropic effects on lipid-lowering property, inflammation, oxidative stress, reduced absorption of advanced glycation end products, bacterial and uremic toxins, and other atherogenic stimuli [16–19]. Short-term treatment of sevelamer elevated serum fetuin-A levels in two studies, which may be related to the decrement of hsCRP after sevelamer treatment [20, 21]. However, the long-term effect of different phosphate binders on serum fetuin-A levels has not been published before.

This post-hoc analysis of a multi-center controlled randomized trial was intended to investigate the long-term effects of sevelamer and calcium containing phosphate binders on serum level of fetuin-A in maintenance HD patients.

## Methods

### Subjects and study design

The original clinical trial was a multi-center, prospective, randomized, open-label, parallel comparison of 48-week treatment of sevelamer (sevelamer HCl, Renagel) versus a calcium-based phosphate binder (calcium carbonate) in maintenance HD patients with hyperphosphatemia. The primary objective was to compare the proportion of patients achieving the National Kidney Foundation’s Kidney Disease Outcomes Quality Initiative (NKF-K/DOQI) recommend treatment goal for maintenance HD patients. It was approved by China medical university and hospital research ethics committees and was registered on *ClinicalTrials.gov* (NCT01755078). The study design and methods also have been reported in detail elsewhere [22]. We conducted a post-hoc analysis to compare the long-term effects of sevelamer versus calcium carbonate on the changes of serum fetuin-A levels. The ethics committees approved consent procedure and the written informed consent was obtained from all patients. This is a per-protocol analysis including only the population who have completed the 48-week treatment.

Adult (age ≥ 45 years) ESRD patients with anuria attending routine HD sessions 3 times per week for at least 3 months with adequate dialysis dose (KT/V >1.2) were evaluated. The different calcium concentrations in dialysate of studied patients included low calcium bath (LCB, calcium concentration 2.5 meq/L) and non- low calcium bath (non-LCB, calcium concentration 3.0 meq/L or 3.5 meq/L). Patients were excluded from the study if they had the following conditions: hypercalcemia (corrected serum total calcium > 10.5 mg/dL) during the 2 weeks of washout period; ALT or AST > 3 times upper normal limit or iPTH > 1000 pg/mL before screening; clinical inflammatory or infectious diseases, gastrointestinal bleeding or any other cause of hospital admission within 3 months before enrollment; thyroid disease, parathyroidectomy, swallowing disorders, gastrectomy or intestinal resection; osteoporosis and concurrently receiving related medications (including bisphosphonates, calcitonin or hormone replacement therapy) and known hypersensitivity to any components of the formulation of the study medications. Commonly used and studied calcium-based phosphate binders include calcium carbonate and calcium acetate. Calcium acetate was not chosen in this study because of the large tablet size manufactured in Taiwan and poor taste, it is unpleasant for seniors to swallow.

At the screening visit, patients who fulfilled the entrance criteria and who gave their written informed consent entered a washout period. They discontinued phosphate binders for two weeks. Patients who had hyperphosphatemia (serum phosphorus > 5.5 mg/dL and ≤ 8.5 mg/dL) during the 2-week washout period were randomized to receive either sevelamer or calcium carbonate for 48 weeks. The randomization schedule was generated using a validated system that automates the random assignment of treatment groups to randomized numbers. All randomized patients attended bi-weekly clinic visits for the first month (week 2 and 4), monthly clinic visits for the subsequent 2 months (week 8 and 12) and tri-monthly clinic visits for the last 9 months (week 24, 36 and 48). The starting dose of the medications was based on the baseline serum phosphate (P) level. All patients in different treatment groups took sevelamer (800mg per tablet) or calcium carbonate (500mg per tablet) three times per day with 1 tablet (5.5 < *P* < 6.5 mg/dL), 2 tablets (6.5 ≦ *P* < 7.5 mg/dL), or 3 tablets (*P* ≧ 7.5 mg/dL). The medications were given with meals and doses were titrated according to a fixed algorithm: increase 1 tablet per meal (if *P* > 5.5 mg/dL), no change (3.5 ≦ *P* ≦ 5.5 mg/dL), or decrease 1 tablet per meal (if *P* < 3.5 mg/dL). If the serum total calcium level rose above 10.5 mg/dL, the investigator reduced the calcium carbonate dosage by 1 tablet per meal to bring the serum calcium below 10.5 mg/dL. The largest daily dose was 12 tablets of sevelamer or calcium carbonate. The total duration of this trial was 50 weeks (*i.e,* 2 weeks washout plus 48 weeks of treatment). The patients were instructed to take trial medication per day and returned the trial medication bottle in next visit. A record of the number of tablets dispensed, taken and returned for each patient was documented on the case report form (CRF). Compliance was assessed from information recorded in the CRF, including trial medication count and starting and ending date of therapy. All patients in the analysis maintained their regular dialysis schedule, dietary habits and prescribed medication for diabetes mellitus (insulin or oral anti-diabetic drugs, except for metformin and glitazones), dyslipidemia (statin), hypertension (anti-hypertension drugs) and secondary hyperparathyroidism (active vitamin D, either alfacalcidol or calcitriol, dosage adjusted by KDOQI guideline) throughout the study period by the physicians in the three centers. Magnesium-containing drugs, other vitamin D analogs and calcimimetics were not prescribed to the patients.

### Measurements

After enrollment, the serum phosphate, total calcium and albumin levels were measured at week 0, 2, 4, 6, 8,12, 24, 36 and 48. The serum ALK-P, LDL-C, hsCRP and iPTH levels were measured at week 0, 12, 24, 36 and 48. The serum fetuin-A levels were measured at week 0 and 48. The serum phosphate, total calcium, lipid profiles, triglyceride, albumin, ALK-P and hsCRP were measured using standard laboratory techniques with an automatic analyzer in the central laboratory. Intact PTH was measured by immunoradiometric assay (Beckman Coulter PTH IRMA).

For the analysis of fetuin-A, the serum was centrifuged and harvested according to standard procedures at 4 °C and frozen immediately afterwards at −80 °C. Serum analysis for fetuin-A was performed in duplicate by a highly sensitive two-site enzyme linked immunoassay (ELISA) (Immunology Consultants Laboratory, Inc. OR, USA.) The interassay and intraassay coefficients of variations were <8 %. Fetuin-A was batch analyzed after completion of the study.

### Statistical analysis

The sample size and power was estimated by G*Power software. We estimated that a sample size of 70 (35 in each one group) with an anticipated dropout rate of 30 % would provide more than 80 % power to detect a significant difference between two independent or dependent means with a two-sided α = 0.05. Shapiro-Wilk test was used to verify the distribution normality of investigated parameters. Normally distributed data were given as mean ± standard deviation, whereas non-normally distributed data were expressed as median (inter-quartile range). Continuous and ordinal data were analyzed using the Student *t* test. Changes in parameters from the baseline data were compared with a paired samples *t* test. The Mann-Whitney *U* test and the Wilcoxon signed-rank test were used for testing nonparametric measurements. Categorical data were analyzed using Fisher’s exact test or the chi-square test. Bivariate relationships were calculated using Pearson or Spearman’s rank correlation coefficient.

## Results

### Baseline characteristics and medications

**Fig. 1:**
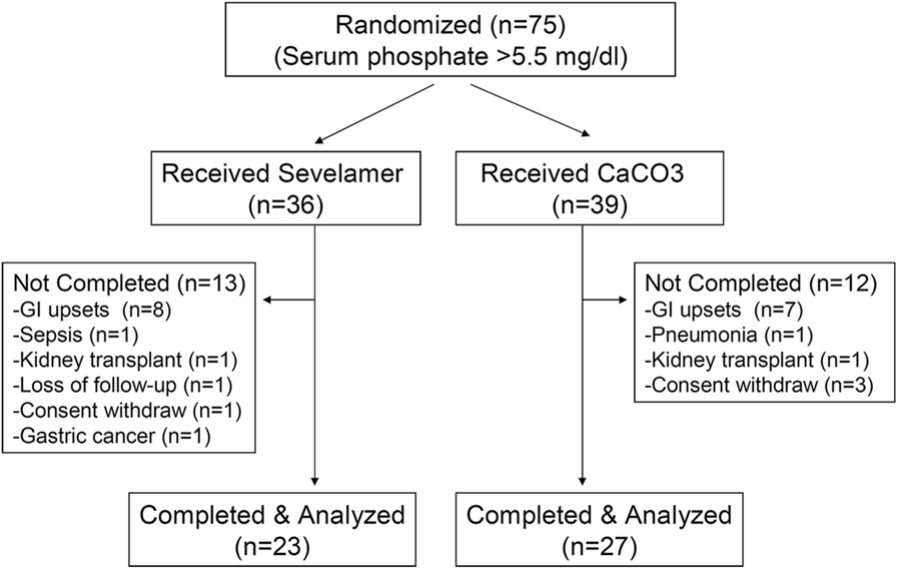
Patient flow chart

In this post-hoc analysis, 75 randomized patients from three large HD centers were included. In the sevelamer group, 13 patients withdrew early (8 patients withdrew in eight weeks because of GI problems, 1 died with sepsis at week 12, 1 received a kidney transplant at week 24, 1 loss to follow-up at week 24, 1 withdrew consent at week 36, and 1 died with gastric cancer at week 36). In the calcium group, 12 patients withdrew early (1 died with pneumonia at week two, 7 patients withdrew in 12 weeks because of GI problems, 2 withdrew their consent at week 24 and week 36, and 1 received a kidney transplant at week 36). Finally, 50 patients completed the 48-week treatment, including 23 patients treated with sevelamer and 27 patients with calcium carbonate. The flow chart was shown in Fig. 1.

Baseline demographic data of 50 completed treatment patients are shown in Table 1. Variables of HD duration and serum levels of iPTH, hsCRP, TG and fetuin-A were not normally distributed. There were no differences between the two groups with respect to age, sex, hemodialysis duration, patients with DM, use of active vitamin D, low calcium dialysate, statin and anti-hypertension drugs. The sevelamer group had lower basal serum phosphate levels (6.54 ± 0.91 vs 7.22 ± 0.98 mg/dL in calcium group, *P* = 0.015) before treatment. No other significant differences of basal serum Ca, iPTH, ALK-P, hsCRP, Hct, Albumin, LDL-C, TG and fetuin-A levels were noted between two groups (Table 1). The baseline serum fetuin-A levels had a significant correlation with HD duration (*ρ* = -0.324, *P* = 0.022) and basal serum albumin levels (*ρ* = 341, *P* = 0.015) (Table 2). For the 25 (33.3 %) patients who withdrew early, the clinical characteristics of them were not different from the 50 analyzed patients in age, sex, hemodialysis duration, number of patients with DM, use of vitamin D, use of low calcium dialysate, use of statin and anti-hypertension drugs, basal serum Ca, P, iPTH, ALK-P, hsCRP, Hct, serum albumin, LDL-C and TG levels (data not shown).

**Table 1:** Baseline characteristics and parameters between two groups

	Sevelamer (*n* = 23)	Calcium Carbonate (*n* = 27)	*P*
Age (yr)	59.61 ± 8.16	56.96 ± 7.72	0.248
Gender (male)	11 (47.83 %)	18 (66.67 %)	0.149
Hemodialysis (yr)	7.00 (5)	5.00 (4)	0.313
DM (no)	9 (39.13 %)	8 (29.63 %)	0.557
Use of vitamin D (no)	9 (39.13 %)	13 (48.14 %)	0.577
Use of vitamin D (months)	77	81	0.775
LCB (no)	7 (30.43 %)	6 (22.22 %)	0.537
Statin (no)	7 (30.43 %)	5 (18.52 %)	0.305
Anti-hypertension drug (no)	16 (69.56 %)	20 (74.07 %)	0.761
Ca (mg/dL)	9.37 ± 0.66	9.37 ± 0.73	0.986
P (mg/dL)	6.54 ± 0.91	7.22 ± 0.98	0.015*
iPTH (pg/mL)	354.70 (332.70)	320.90 (371.8)	0.633
ALK-P (IU/L)	82.17 ± 32.71	65.67 ± 23.70	0.061
HsCRP (mg/L)	2.3 (5.8)	3.10 (6.1)	0.697
Hct (%)	33.10 ± 3.87	32.49 ± 3.11	0.549
Albumin (g/L)	39.92 ± 3.13	39.81 ± 2.86	0.866
TG (mg/dL)	98.00 (86.00)	113.00 (127.00)	0.459
LDL-C (mg/dL)	108.96 ± 30.10	112.93 ± 33.46	0.661
Fetuin-A (ug/mL)	210.61 (104.73)	203.95 (107.87)	0.961

Data are mean ± standard deviation or median (interquartile range, IQR)

Vitamin D: active vitamin D, either alfacalcidol or calcitriol

LCB: low calcium bath (Ca 2.5 meq/L); non-LCB: Ca 3.0 meq/L or 3.5 meq/L

*P* < 0.05*

**Table 2:** Correlation of baseline serum fetuin-A and other parameters

	Fetuin-A: Coefficient ρ (*P*)
Age (yr)	−0.209 (0.145)
HD duration (yr)	−0.324 (0.022*)
DM or not	−0.116 (0.424)
Ca	0.100 (0.491)
P	−0.016 (0.915)
iPTH	0.105 (0.467)
ALK-P	−0.40 (0.783)
HsCRP	−0.135 (0.349)
Albumin	0.341 (0.015*)
LDL-C	0.111 (0.443)
TG	−0.011 (0.937)

*P* < 0.05*

The average daily dose of study medication was 7.81 ± 3.22 tablets (6248 ± 2576 mg) in sevelamer group and 6.52 ± 2.61 tablets (3260 ± 1305 mg) in calcium carbonate group. The incidence of combined adverse events was similar between sevelamer and calcium carbonate group. The most commonly reported adverse event in sevelamer group was upper abdominal pain (8.0 %, vs. 0 %, *P < 0.001*). Constipation was more common in the calcium carbonate group (7.3 %, vs. 4.5 %, *P = 0.022*). There were no cardiovascular events in either study group during the follow-up period.

### Comparison of serum fetuin-A level and other parameters before and after 48-week treatment

In the sevelamer group, there were significant changes after the 48-week treatment with increased serum calcium, decreased serum phosphate, increased ALK-P, decreased hsCRP, decreased LDL-C and decreased fetuin-A levels (from 210.61 (104.73) to 153.85 (38.64) ug/dl, *P* = 0.003) (Table 3). In the calcium group, there were significant changes after the 48-week treatment with increased serum calcium, decreased phosphate, decreased iPTH and decreased fetuin-A levels (from 203.95 (107.87) to 170.90 (58.02) ug/mL, *P* =0.002) (Table 3).

**Table 3:** Comparison of parameters before and after treatment in each group

	Sevelamer (*n* = 23)			Calcium Carbonate (*n* = 27)		
	BT	AT	*P*	BT	AT	*P*
Ca (mg/dL)	9.37 ± 0.66	9.63 ± 0.80	0.038*	9.37 ± 0.73	10.17 ± 0.90	0.000*
P (mg/dL)	6.54 ± 0.91	5.07 ± 0.85	0.000*	7.22 ± 0.98	5.70 ± 1.01	0.000*
iPTH (pg/mL)	354.70 (332.70)	329.60 (319.80)	0.903	320.90 (371.8)	165.20 (405.60)	0.001*
ALK-P (IU/L)	82.17 ± 32.71	112.43 ± 43.21	0.004*	65.67 ± 23.70	64.41 ± 23.13	0.609
HsCRP (mg/L)	2.3 (5.8)	1.6 (3.1)	0.003*	3.10 (6.1)	1.9 (6.6)	0.866
Hct (%)	33.10 ± 3.87	32.80 ± 3.72	0.728	32.49 ± 3.11	32.49 ± 4.58	0.996
Albumin (g/L)	39.92 ± 3.13	39.96 ± 3.02	1.000	39.81 ± 2.86	40.78 ± 2.95	0.062
TG (mg/dL)	98.00 (86.00)	96.00 (103)	0.891	113.00 (127.00)	103.00 (65)	0.343
LDL-C (mg/dL)	108.96 ± 30.10	63.91 ± 20.55	0.000*	112.93 ± 33.46	105.19 ± 30.93	0.157
Fetuin-A (ug/mL)	210.61 (104.73)	153.85 (38.64)	0.003*	203.95 (107.87)	170.90 (58.02)	0.002*

*BT* before treatment, *AT* after treatment

Data are mean ± standard deviation or median (interquartile range, IQR)

*P* < 0.05*

### Comparison of changes in serum fetuin-A and other parameters after 48-week treatment between two groups

After the 48-week treatment, the sevelamer group had less serum calcium increment (0.26 ± 0.55 vs. 0.80 ± 0.93 mmol/L, *P* = 0.015), less iPTH decrement (12.10 (216.60) vs. -137.10 (255.70) pg/mL, *P* = 0.026), more ALK-P increment (30.26 ± 45.78 vs. -2.26 ± 22.69 IU/L, *P* = 0.004), more hsCRP decrement (−0.9 (2.7) vs. 0.1(3.8) mg/L, *P* = 0.025) and more LDL-C decrement (1.16 ± 0.73 vs. 0.20 ± 0.71 mmol/L, *P* < 0.0001). There was no significant difference in the serum fetuin-A decrement between two treatment groups (sevelamer group −49.44 (113.78) vs. calcium carbonate group −38.21 (78.51) ug/mL, *P* = 0.345) (Table 4).

**Table 4:** Comparison of the changes of parameters before and after treatment between two groups

	Sevelamer (*n* = 23)	Calcium Carbonate (*n* = 27)	*P*
Δ Ca (mg/dL)	0.26 ± 0.55	0.80 ± 0.93	0.015*
Δ P (mg/dL)	−1.47 ± 0.98	−1.53 ± 1.27	0.871
Δ iPTH (pg/mL)	12.10 (216.60)	−137.10 (255.70)	0.026*
Δ ALK-P (IU/L)	30.26 ± 45.78	−2.26 ± 22.69	0.004*
Δ HsCRP (mg/L)	−0.9 (2.7)	0.1 (3.8)	0.025*
Δ Hct (%)	−0.30 ± 4.00	−0.00 ± 3.39	0.785
Δ Albumin (g/L)	0.00 ± 2.72	0.82 ± 2.31	0.241
Δ TG (mg/dL)	−11.00 (70)	−13.00 (93)	0.346
Δ LDL-C (mg/dL)	−45.04 ± 28.31	−7.74 ± 27.59	0.000*
Δ Fetuin-A (ug/mL)	−49.44 (113.78)	−38.21 (78.51)	0.345

Δ Ca: the change in serum calcium concentration

Data are mean ± standard deviation or median (interquartile range, IQR)

*P* < 0.05*

### Correlations of the changes in serum fetuin-A and related parameters

We further analyzed the correlations of fetuin-A decrement with related parameters in 50 studied patients. Bivariate correlation analysis (Table 5) showed the decrement in serum fetuin-A (Δfetuin-A) levels was associated with the changes in serum calcium (ΔCa) (*ρ* = −0.230, *P* = 0.040), iPTH (ΔiPTH) (*ρ* = 0.306, *P* = 0.031) and albumin (Δalbumin) levels (*ρ* = 0.408, *P* = 0.003), not associated with sevelamer use, changes in serum phosphate (ΔP) and hsCRP (ΔhsCRP) levels.

**Table 5:** Correlation of Δ Fetuin-A and changes of related parameters

	Fetuin-A: Coefficient ρ (*P*)
Sevelamer or not	−0.135 (0.350)
Vitamin D or not	0.017 (0.908)
LCB or not	0.115 (0.425)
Δ Ca	−0.230 (0.040*)
Δ P	−0.091 (0.531)
Δ iPTH	0.306 (0.031*)
Δ ALK-P	0.145 (0.314)
Δ hsCRP	−0.085 (0.558)
Δ Albumin	0.408 (0.003*)
Δ TG	−0.113 (0.434)
Δ LDL-C	0.132 (0.363)

Δ Fetuin-A: the change in serum Fetuin-A concentration

Vitamin D: active vitamin D, either alfacalcidol or calcitriol

LCB: low calcium bath (Ca 2.5 meq/L); non-LCB: Ca 3.0 meq/L or 3.5 meq/L

*P* < 0.05*

## Discussion

To the best of our knowledge, this is the first study to evaluate factors associated with serum fetuin-A concentrations after long-term use of different phosphate binders in maintenance HD patients. After 48-week sevelamer or calcium carbonate treatment, both groups of maintenance HD patients had lower serum fetuin-A levels. Our study first pointed out that the decreased serum fetuin-A levels was associated with serum calcium increment, iPTH decrement and albumin decrement.

Fetuin-A binds to serum calcium and phosphate, forming small calciprotein particles that are presumably removed through the reticuloendothelial system and normal kidney [10]. Fetuin-A is considered a host defense to clean unwanted calcium and phosphate in serum and prevent undesirable calcification in the circulation [23]. Fetuin-A-deficient mice on the calcification-prone genetic background DBA/2 develop severe calcification in most soft tissues [24]. Fetuin-A levels in hemodialysis patients showed a positive association with serum albumin and a reverse association with hsCRP and dialysis duration [25, 26]. In our study, the baseline serum fetuin-A levels had a significant positive correlation with serum albumin levels and a negative correlation with HD duration. The finding is comparable with previous studies [25, 26]. The results suggest patients with longer HD duration and lower serum albumin levels will have lower serum fetuin-A concentrations.

Previously, one short-term (8 weeks) study in CKD stage 4 patients and another short-term (8 weeks) study in chronic HD patients both showed sevelamer treatment elevated the serum fetuin-A levels [20, 21]. The elevation of serum fetuin-A may be related to the decrement of hsCRP after sevelamer treatment [20, 21]. However, our study did not confirm the decreased hsCRP- related fetuin-A increment after long-term sevelamer treatment. The decreased serum hsCRP levels was also noted after long-term sevelamer treatment. Our long-term study revealed the serum fetuin-A decrement was associated with the serum calcium increments, iPTH decrements and albumin decrements, not related to the changes of serum hsCRP levels. As we know, serum fetuin-A levels has a negative correlation with HD duration. Both the increment of serum calcium levels and the time effect of 48-week HD duration may account for the negative finding of fetuin-A increment after long-term sevelamer treatment. The cause of mild elevation of serum calcium levels in the sevelamer group may be due to the calcium loading from the calcium-containing dialysate bath. Our findings also implied that high calcium bath and long-term calcium-based phosphate binder had worse impacts on serum fetuin-A levels, especially in patients with increased serum calcium and decreased serum iPTH levels.

This study had a number of limitations. First, the number of studied patients was small and multivariate analysis was not further performed. Second, we did not take an intermediate measurement of fetuin-A level at week 24 of the study. Third, although the number of patients and the duration of active vitamin D treatment were not different between the two groups, we did not measure the serum vitamin D3 levels of these patients. The effect of vitamin D therapy on serum fetuin-A levels is still conflicting [27, 28].

## Conclusions

Our study is the first to disclose the long-term effect of different phosphate binders on serum fetuin-A levels in chronic HD patients. After long-term sevelamer or calcium carbonate treatment, both groups of maintenance HD patients had lower serum fetuin-A levels. Serum levels of increased calcium, decreased iPTH and decreased albumin were associated with the serum fetuin-A decrement.

## Abbreviations

CKD-MBD: Chronic kidney diseases- mineral and bone disorders
CPPs: Calciprotein particles
CVD: Cardiovascular disease
ELISA: Enzyme linked immunoassay
ESRD: End-stage renal disease
HD: Hemodialysis
hsCRP: High sensitivity C-reactive protein
iPTH: Intact parathyroid hormone
LDL-C: Low density lipoprotein cholesterol
NKF-K/DOQI: National Kidney Foundation’s Kidney Disease Outcomes Quality Initiative
